# Supplementary material for: Mutations in PCYT2 disrupt etherlipid biosynthesis and cause a complex hereditary spastic paraplegia
Source: Brain. 2019 Oct 22;142(11):3382–97. doi: 10.1093/brain/awz291 (PMC6821184; doi:10.1093/brain/awz291)
Supplement: awz291_Supplementary_Data [file awz291_supplementary_data.zip › awz291-Suppl_data/Supplementary_Data4.zip!/Supplementary information 3 - fibroblast lipidomics.zip!/Fibroblast lipidomics/Patient1 vs Patient2/Statistical plots and data/Volcano plot_alternative.pdf]

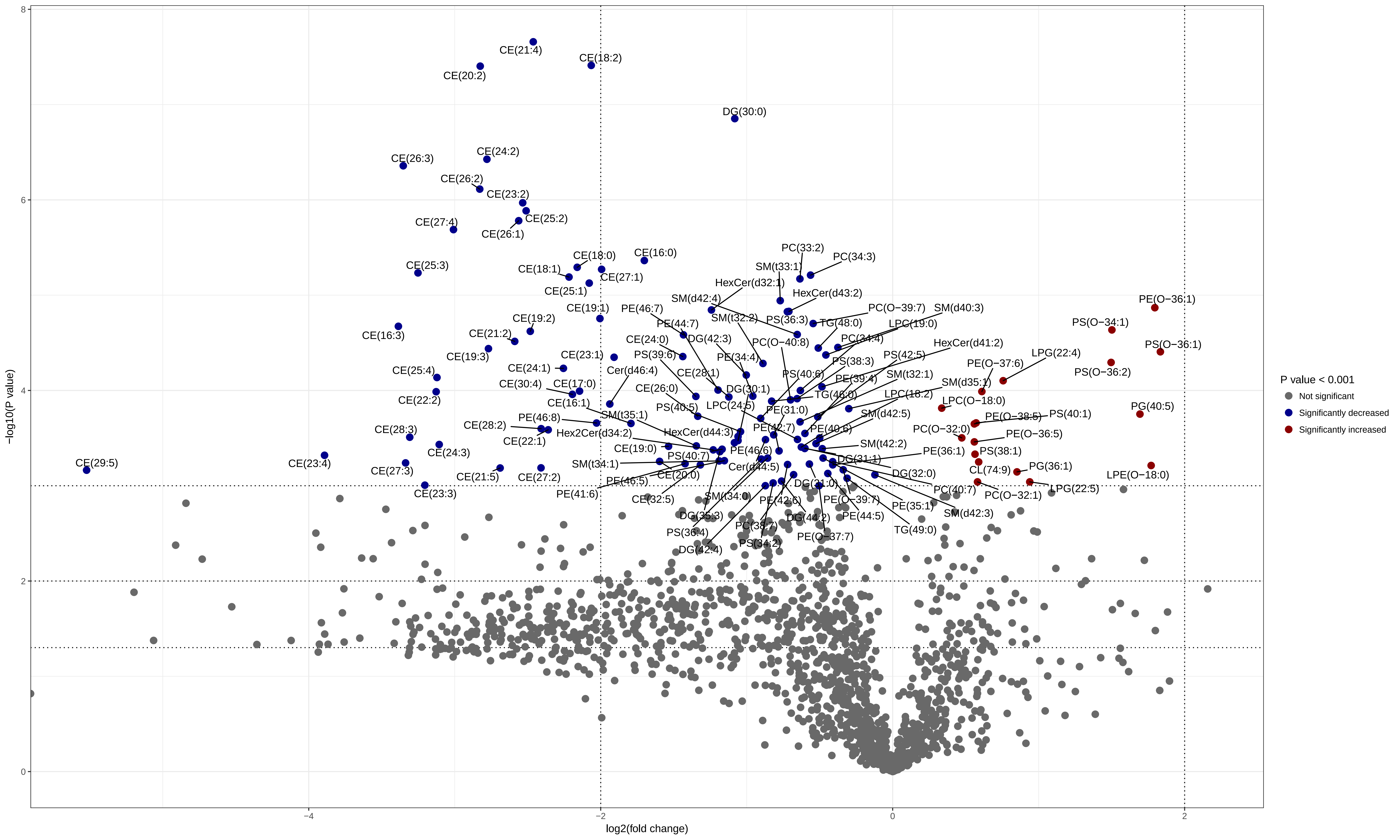

CE(21:4)
CE(18:2)
CE(20:2)
DG(30:0)
CE(26:3)
CE(24:2)
CE(26:2)
CE(23:2)
CE(27:4)
CE(25:2)
CE(26:1)
PC(33:2)
CE(18:0)
CE(16:0)
PC(34:3)
CE(25:3)
CE(18:1)
SM(t33:1)
CE(27:1)
HexCer(d32:1)
CE(25:1)
HexCer(d43:2)
PE(O−36:1)
SM(d42:4)
CE(19:1)
PE(46:7)
PC(O−39:7)
SM(d40:3)
CE(19:2)
SM(t32:2)
PS(36:3)
TG(48:0)
PS(O−34:1)
PE(44:7)
LPC(19:0)
CE(16:3)
CE(21:2)
CE(24:0)
DG(42:3)
PC(O−40:8)
PC(34:4)
HexCer(d41:2)
PS(O−36:1)
CE(19:3)
CE(23:1)
PS(39:6)
PE(34:4)
PS(38:3)
PS(42:5)
LPG(22:4)
CE(24:1)
Cer(d46:4)
CE(28:1)
PE(O−37:6)
CE(25:4)
PS(40:6)
PE(39:4)
SM(t32:1)
PS(O−36:2)
CE(30:4)
CE(17:0)
DG(30:1)
SM(d35:1)
CE(26:0)
TG(46:0)
LPC(18:2)
CE(22:2)
CE(16:1)
PS(40:5)
LPC(24:5)
LPC(O−18:0)
PE(31:0)
PG(40:5)
SM(t35:1)
SM(d42:5)
PE(46:8)
CE(28:2)
PE(O−38:5)
PS(40:1)
CE(28:3)
PE(42:7)
PE(40:6)
Hex2Cer(d34:2)
HexCer(d44:3)
PC(O−32:0)
CE(22:1)
PE(O−36:5)
SM(t42:2)
CE(24:3)
CE(19:0)
PE(46:6)
PE(36:1)
PS(38:1)
CE(29:5)
CE(23:4)
PS(40:7)
DG(31:1)
SM(t34:1)
Cer(d44:5)
CL(74:9)
PG(36:1)
CE(27:3)
CE(20:0)
DG(32:0)
LPE(O−18:0)
CE(21:5)
CE(27:2)
PE(46:5)
DG(31:0)
PC(40:7)
CE(23:3)
PC(O−32:1)
LPG(22:5)
PE(41:6)
SM(34:0)
CE(32:5)
PE(42:6)
PE(O−39:7)
PE(35:1)
SM(d42:3)
DG(35:3)
DG(44:2)
PE(44:5)
PC(38:7)
TG(49:0)
PS(36:4)
PE(O−37:7)
PS(34:2)
DG(42:4)
P value < 0.001
Not significant
Significantly decreased
Significantly increased
−log10(P value)
log2(fold change)
8
6
4
2
0
−4
−2
0
2
